# Photodynamic therapy for *Cutibacterium acnes* decolonization of the shoulder dermis

**DOI:** 10.1177/17585732231192856

**Published:** 2023-08-07

**Authors:** John G Horneff, Alayna Vaughan, Manan Patel, Thema Nicholson, Serge Tzeuton, Mark Lazarus, Surena Namdari, Joseph A Abboud

**Affiliations:** 1 6572 Department of Orthopaedic Surgery, University of Pennsylvania, Philadelphia, PA, USA; 2 Division of Shoulder and Elbow Surgery, Rothman Orthopaedics, Thomas Jefferson University, Philadelphia, PA, USA

**Keywords:** *Cutibacterium acnes*, shoulder arthroscopy, shoulder infection, *C. acnes*, blue light therapy, bacterial colonization

## Abstract

**Introduction:**

*Cutibacterium acnes (C. acnes)* is a common source of infection in shoulder surgery. 5-Aminolevulinic acid (5-ALA) is a naturally occurring metabolite of *C. acnes* that creates an exothermic reaction when activated by blue light. The purpose of this study was to evaluate the efficacy of preoperative photodynamic therapy using topical 5-ALA to decrease *C. acnes* colonization.

**Methods:**

Patients were randomized to receive topical 5-ALA skin solution activated by blue light photodynamic therapy or standard of care preoperative skin preparation. Prior to skin incision, two punch biopsy specimens were taken from the portal sites and were incubated for 13 days. Culture positivity rate, days until positive, and semiquantitative growth classification were analyzed.

**Results:**

Fifty patients undergoing arthroscopic shoulder surgery were randomized. The overall positive culture rate was 54%. All cultures were positive for *C. acnes* except for one. Sixty-four percent of standard preparation patients and 44% of investigational group patients had at least one positive culture for *C. acnes*. There was no significant difference between groups in patients with at least one positive *C. acnes* culture (*p* = 0.49).

**Conclusion:**

The use of photodynamic therapy undergoing arthroscopic shoulder surgery did not significantly reduce colonization of *C. acnes* as compared to standard preparation.

**Level of Evidence:**

Level II.

## Introduction

*Cutibacterium acnes* (*C. acnes*), formerly known as *Propionibacterium acnes*, has been recognized as a significant causal pathogen in orthopedic surgery infections over the last decade. Patients with a *C. acnes* infection of the shoulder can suffer from indolent pain, septic arthritis, and even arthroplasty failure.^1,2^ The shoulder axilla is known to have increased rates of *C. acnes* colonization compared to hip and knee,^1,3^ increasing the potential for surgical site infection at the shoulder. Although its clinical importance remains unknown, the high rate of colonization around the shoulder and its potentially indolent presentation makes *C. acnes* a substantial infectious concern when performing shoulder surgery. Thus, reducing the bacterial burden of *C. acnes* has been postulated to reduce deep colonization and infection rates after shoulder surgery. To address this problem, many researchers have examined the use of various topical solutions as well as more systemic treatments, in the hope of decreasing the bacterial burden of *C. acnes* in shoulder surgery patients. Peroxide-based solutions, topical antibiotics, oral antibiotic prophylaxis, and various surgical instrument handling techniques have all been examined with various degrees of success in preventing deep tissue infections.^4–10^

*C. acnes* has been further studied as a causal bacterium in acne vulgaris, showing that more than 40% of *C. acnes* is resistant to topical and oral anti-acne antibiotic treatments.^7,10–13^ This rise in antibiotic resistance has led to increased research in the eradication of *C. acnes* through photodynamic therapy (PDT).^11,14^ Through its metabolic pathways, *C. acnes* is known to produce high levels of intracellular porphyrins which creates reactive oxygen species (ROS). When exposed to light, these ROS induce phototoxic reactions which can lead to bacterial destruction.^15^ This destruction of bacterial cells by these porphyrins has been found to be very efficient.^11^ The bacterial destruction by PDT can be further augmented by 5-aminolevulinic acid (ALA), a naturally occurring metabolite in the synthesis pathway of cellular heme production.^11^ Adding ALA to bacteria encourages porphyrin production. When these porphyrins are illuminated with blue light at an emission peak of 407 to 420nm, these metabolites become exothermic and cause internal destruction of the bacterial cells.^11^ This therapy does not cause any damage to the mammalian cells, however, which makes PDT safe for human skin treatment.^11^

The purpose of this prospective randomized controlled trial was to evaluate the efficacy of photodynamic activation of an ALA solution as an addition to standard sterile preparation for shoulder surgery. We hypothesized that preparation with ALA solution activated by blue light therapy in addition to standard sterile preparation would decrease the incidence of positive dermal cultures of *C. acnes* among male patients undergoing arthroscopic shoulder surgery.

## Materials and methods

This study was a prospective, randomized controlled trial, performed at a tertiary referral practice by four shoulder and elbow–trained subspecialty attending surgeons between August 2019 and January 2022. Following institutional review board approval, patients were recruited preoperatively for study participation and enrolled after patient informed consent.

Inclusion criteria were male patients, 18 years or older, undergoing shoulder arthroscopy. Exclusion criteria consisted of patients with active acne at the surgical site or history of psoriatic or eczematous lesions about the shoulder girdle, a history of postsurgical shoulder infection, those that had taken any antibiotics in the past month, or those with allergies to any components of the surgical preparation. Enrolled patients were then randomized to either the control group (standard preoperative sterile preparation) or the study group (addition of ALA HCl topical solution prior to the preoperative preparation).

**Figure 1.**
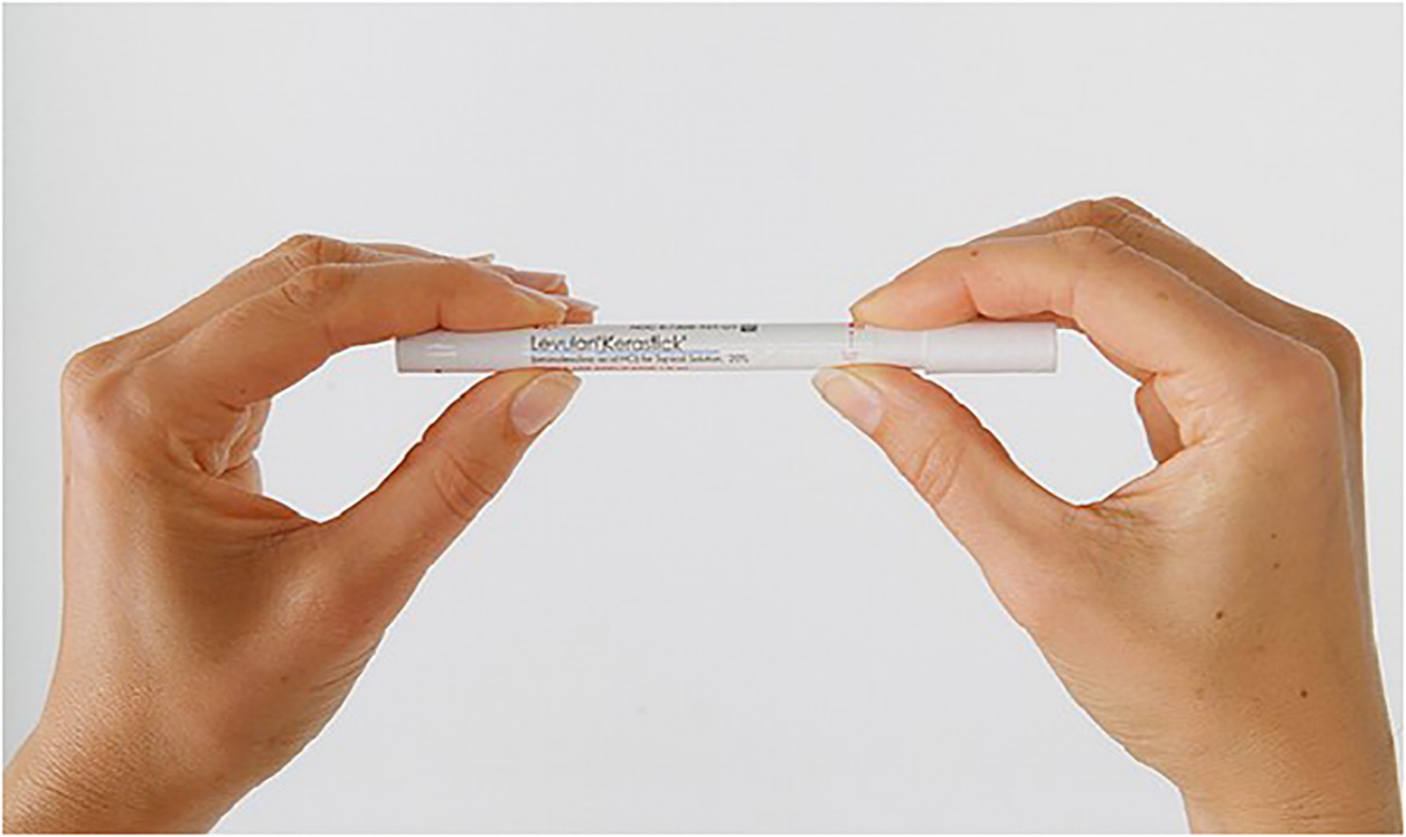
Levulan Kerastick applicator. Patients are instructed to crush the bottom ampule containing the solution vehicle by applying finger pressure to the cardboard sleeve, shake the applicator for 30 s to dissolve the drug powder, then place the dry tip to the operative shoulder.

Patients in the study group were given written and visual instructions on applying ALA HCl (Levulan Kerastick, DUSA Pharmaceuticals Inc., Billerica, MA, USA) topical solution 20% sticks in the region of the operative shoulder and axilla. Patients were instructed to apply the topical solution after showering the morning of surgery (Figure 1). Preoperatively, the patients underwent photoactivation of the ALA HCl while in the preoperative holding area. A blue light photodynamic therapy illuminator was applied to the shoulder for 1000 s (16 min, 40 s), which is the necessary time of exposure to provide the 10J/cm^2^ light dose to activate the solution.

Once this was completed, all patients underwent the standard preoperative preparation beginning with hair removal in the region of the planned arthroscopic portals removed with a battery-powered hair clipper in the preoperative waiting area. The first skin cleanse was done with 2% chlorhexidine gluconate cloths (Sage Products, Cary, IL, USA). Routine perioperative antibiotics were administered within 1 h of surgery, either weight-based cefazolin or vancomycin for those with a cephalosporin allergy. In the operating room, the upper extremity, including the shoulder and axilla, was cleansed with saturated 7.5% povidone-iodine solution surgical scrub brushes (Betadine Surgical Scrub; Purdue Pharma, Stamford, CT, USA) and then wiped clean with a sterile towel. The remainder of the preparation was uniform for both groups. Sterile preparation with five 3% hydrogen peroxide-soaked gauzes followed by two 26 mL stick applicators with 2% chlorhexidine gluconate and 70% isopropyl alcohol formulation (ChloraPrep; Cardinal Health, Dublin, OH, USA) were used to cleanse the shoulder, axilla, and arm. Surgical draping was then performed in the standard sterile manner for an arthroscopic shoulder surgery.

Following surgical timeout and prior to incision for surgical start, a 3 mm punch biopsy was obtained from the anterior and posterior arthroscopic portal site of all enrolled patients (Disposable Biopsy Punch; Robbins Instruments, Chatham, NJ, USA). Biopsy samples were transferred immediately via sterile forceps into single sterile specimen containers and sent directly to the lab for culture. The laboratory was blinded to sample cohorts. Anaerobic and aerobic culture substrates were used as growth mediums, as it has been reported that *C. acnes* can grow on either substrate.^16^ Biopsy cultures were held for 13 days.^17,18^

### Statistical analysis methods

Based on the assumption that a 30% difference in positive culture rates would be clinically significant, the number of patients required to achieve 80% power at alpha = 0.05 was 45 patients per group. This 30% difference was slightly modified from the 35% decrease in positive culture rate observed by Lee et al.^5^ An interim analysis would be performed to at mid-term enrollment. The intention was to possibly terminate the trial early or adjust the sample size. The data was broken down first descriptively to understand the overall distributions. Normality was assessed by performing Shapiro-Wilks test. Mann-Whitney U tests were used to calculate p values for continuous data and chi-square or Fisher's exact tests were used to calculate p values for categorical data. Significance was determined at p value <0.05.

## Results

Fifty-one patients undergoing arthroscopic shoulder surgery were enrolled in the study after informed consent was obtained. Patients were randomized into the ALA group consisting of 25 patients and a control group consisting of 26 patients. A total of 65 patients were approached for participation. Fourteen patients were excluded from the study due to surgery cancelation or study protocol deviations. At an interim analysis, a neutral effect was present in the light therapy group and it became increasingly unlikely that a positive, clinically relevant effect would be demonstrated by the end of the trial. As a result, the study was terminated at mid-term. There was no significant difference between the groups in age, body mass index, or Charlson comorbidity index (Table 1). Three patients had prior history of shoulder surgery. There were no severe adverse skin reactions caused by the topical solution.

**Table 1.** Patient demographics.

Variable	Standard prep	Range	ALA prep with photodynamic therapy	Range	p Value
Age (years; mean ± SD)	52.3 ± 12.9	18-74	58.1 ± 8.1	40–77	0.13
BMI (mean ± SD)	30.1 ± 6.2	20.8–46	29.8 ± 5.9	23.1–48.7	0.84
CCI (mean ± SD)	1.4 ± 1.5	0–7	51.6 ± 1.1	0–5	0.22

ALA: aminolevulinic acid; BMI: body mass index; CCI: Charlson comorbidity index; SD: standard deviation.

And 54.9% (28 patients) had at least one positive culture in the entire cohort, 61.5% (16 patients) of standard preparation patients, and 48.0% (12 patients) of ALA group. All positive cultures grew *C. acnes* except for one culture which grew *Staphylococcus saccharolyticus.* Overall, in patients who had at least one positive culture, there was no significant difference between the two groups (p = 0.49). Cultures were positive at a mean of 6.5 days in the standard prep group and 5.8 days in the ALA prep group. There was no significant difference in time to positive culture (p = 0.32). Both anterior and posterior portals were positive in 32% of the ALA prep group. There was no significant difference in positive culture rate between the control group following normal surgical preparation protocol and the ALA prep group utilizing topical ointment with blue light photoactivation in either anterior or posterior portals as well as both portals (p = .88, p = 0.85, p = 0.93) (Table 2).

**Table 2.** Culture results between control and photoactivation groups as determined by portal site location.

Group	Anterior portal *C. acnes* positive rate % (count)	Days until culture positive (mean ± SD)	Posterior portal *C. acnes* positive rate % (count)	Days until culture positive (mean ± SD)	Both portals *C. acnes* positive rate
Standard prep	50.0% (13)	6.2 ± 1.3	38.5% (10)	6.6 ± 1.8	26.9% (7)
ALA prep with photodynamic therapy	44.0% (11)	5.9 ± 2.0	32.0% (8)	5.1 ± 0.8	32.0% (8)
p Value	0.88	0.59	0.85	0.07	0.93

ALA: aminolevulinic acid; *C. Acnes: Cutibacterium acnes*; SD: standard deviation.

Lastly, cultures were categorically sorted into moderate growth, light growth, and very light growth (Tables 3 and 4). There was no significant difference in the bacterial abundance for positive cultures between groups.

**Table 3.** Bacterial abundance in positive cultures in anterior portal.

Group	Total number of positive cultures	Very light growth	Light growth	Moderate growth
Standard prep	12	50.0% (6)	25.0% (3)	25.0% (3)
ALA prep with photodynamic therapy	10	30.0% (3)	30.0% (3)	40.0% (4)

p = 0.66.

ALA: aminolevulinic acid.

**Table 4.** Bacterial abundance in positive cultures in posterior portal.

Group	Total number of positive cultures	Very light growth	Light growth	Moderate growth
Standard prep	10	50.0% (5)	20.0% (2)	30.0% (3)
ALA prep with photodynamic therapy	8	12.5% (1)	37.5% (3)	50.0% (4)

p = 0.29.

ALA: aminolevulinic acid.

## Discussion

*C. acnes* is gram-positive anaerobic bacillus that lives deep in the hair follicles and sebaceous pores of the dermal layer of the skin in addition to the skin surface. The bacteria survive on the metabolic byproducts found in the oily sebum of the sebaceous gland. The neck, axilla, and chest have a much higher concentration of these follicles and pores which leads to a higher density of colonization of the bacteria in these regions, especially in males. The deeper location of the bacteria in the skin layer makes it difficult to eradicate with the use of the standard surgical preparations. This study examined the use of photodynamic therapy to help decrease the colonization of *C. acnes* on the skin of the shoulder given its successful use in treating acne vulgaris.

Since infection with *C. acnes* often proves difficult to diagnose and treat, much of the recent orthopedic literature has looked toward prevention of infection through various prophylactic measures. Unfortunately, the standard surgical preparation solutions that are used in orthopedic surgery are ineffective against *C. acnes*. A randomized controlled trial compared three commercially available surgical-site preparation solutions including povidone-iodine, ChloraPrep (2% chlorhexidine gluconate and 70% isopropyl alcohol), and DuraPrep (0.7% iodophor and 74% isopropyl alcohol). The study found that no solution was superior in eliminating *C. acnes*.^19^ Another study demonstrated that 70% of healthy male subjects had dermal punch biopsies that were positive for *C. acnes* even after standard skin preparation with ChloraPrep.^5^

In an effort to penetrate into the deeper layers of the skin, peroxide-based solutions such as benzyl peroxide and hydrogen peroxide have been examined as they are known to penetrate the deeper layers of the skin. Kolakowski et al. demonstrated a significant decrease in *C. acnes* cultures with the use of a 3-day course of topical 5% benzoyl peroxide applied around the shoulder girdle.^20^ Authors of our study previously demonstrated success a significant reduction of *C. acnes* in dermal biopsies that were treated preoperatively with 3% hydrogen peroxide in addition to standard preparation.^7^ Other studies examining the routine use of hydrogen peroxide in surgical preparation have not found any significant impact on decreasing *C. acnes* contamination.^21^ Interestingly, all the patients in our study were treated with 3% hydrogen peroxide as part of their standard surgical preparation and over half (54.9%) had at least one positive culture for *C. acnes.*

The notion of using photodynamic therapy to decrease the bacterial burden of *C. acnes* around the shoulder was inspired by dermatological literature looking at the use of ALA photoactivation to treat acne vulgaris. The first described efficacy study of PDT in acne vulgaris was performed on 22 patients with ALA application to their backs. Improvement was seen at 3 weeks with complete destruction or 45% in sebaceous gland size as the acne clinically improved.^22^ Ashkenazi et al. examined the impact of ALA augmentation of PDT eradication of *C. acnes* bacteria grown on agar plates. Bacteria were allowed to grow in clostridial broth both with and without ALA at various incubation intervals. When exposed to blue light at a wavelength, the viable counts of *C. acnes* dropped off significantly.^11^ The longer the bacteria were incubated in the ALA solution prior to illumination, the greater the effect of the PDT treatment. However, even just 24 h of ALA exposure prior to illumination was enough to decrease the viable number of *C. acnes* by one to two orders of magnitude.^11^

Unfortunately, the results of our study did not demonstrate similar reductions in *C. acnes* colonization. At an interim analysis, a neutral effect was present in the light therapy group and it became increasingly unlikely that a positive, clinically relevant effect would be demonstrated by the end of the trial. As a result of this analysis, the study was terminated early as the side effect profile of the therapy appeared to outweigh any clinical benefit. One concern at the time of performing this study was the possible negative side effects of photodynamic therapy in the surgical field of the shoulder. In consulting with the manufacturers of the Levulan Kersticks, the concern for possible skin peeling at the site of application was noted. Although a relatively rare reaction, it was noted that an increased duration of time between the application of the Levulan Kerastick solution and the photoactivation using the blue light significantly elevated the risk of such a skin peeling reaction. As such, we decided to only have the solution applied on the skin for a few hours prior to photoactivation with the blue light therapy. Given this shorter duration for dermal penetrance, it is possible that the therapeutic effects of *C. acnes* eradication were impacted negatively. However, we did not want to risk exposing our patients’ fresh surgical incisions to any compromised skin reactions.

In addition to this concern, this study had multiple weaknesses. There is a potential for contamination with transport of the tissue samples. Multiple previous studies have shown false positive rates on *C. acnes* growth in even sterile swabs cultured at the time of the surgeries. Additionally, although we had the patients sit under the blue light for the recommended duration of activation, there was possible variation in the total time they had placed the preparation on the skin prior to light activation given that they were told to only apply the solution prior to arrival. As such, the variability in skin penetrance was increased with the inability to standardize the time between solution application and photoactivation. In future studies, we would strongly recommend application of the skin preparation under direct surveillance of the study investigators to ensure proper technique and duration. Further studies are required to balance the effective duration of skin preparation and minimization of potential side effects.

Despite the promise of photoactivation therapy, we are unable to recommend its usage for decreasing *C. acnes* colonization around the shoulder currently. Our results demonstrated no significant difference with the use of photodynamic light therapy compared to a standard preparation demonstrated in reducing the skin burden of *C. acnes*. We performed a post hoc power analysis and we found that the comparison of cultures had a power of 0.41. This does indicate that we were underpowered. However, if we wanted to reach full power of 0.8, we would have needed a total of 126 patients with 63 patients in each group. Given the amount of patient cooperation as well as the inefficiency and cost of its application prior to shoulder surgery, the addition of light therapy remains investigational only. Although our study also noted relatively high percentage of patients in both groups with positive cultures despite the use of hydrogen peroxide as part of the preparation, its ease of use and low cost remain worthwhile in skin preparation. Further research remains warranted in understanding how to safely apply photoactivation therapy as a prophylactic source of reducing *C. acnes* colonization.

## Conclusion

*C. acnes* remains a concerning organism for deep infections about the shoulder. Various methods to reduce the dermal burden of *C. acnes* to prevent deep infection have demonstrated mixed results. The results of this study suggest that the use of photodynamic therapy to activate exothermic metabolites of *C. acnes* is not helpful in reducing bacterial burden compared to standard surgical preparation alone. Further studies examining the use of photodynamic therapy as an adjuvant to reducing *C. acnes* colonization of the shoulder dermis are warranted.
